# Physical activity and vascular calcification in maintenance hemodialysis: the mediating role of irisin

**DOI:** 10.3389/fendo.2026.1852364

**Published:** 2026-07-06

**Authors:** Huilan Li, Zhengjia Fan, Aihua Zhang

**Affiliations:** 1 Department of Nephrology, Xuanwu Hospital, Capital Medical University, Beijing, China; 2 National Clinical Research Center for Geriatric Diseases, Xuanwu Hospital, Capital Medical University, Beijing, China

**Keywords:** irisin, maintenance hemodialysis, mediation analysis, physical activity, vascular calcification

## Abstract

**Introduction:**

Vascular calcification (VC) is a major determinant of cardiovascular mortality in patients undergoing maintenance hemodialysis (MHD). Although physical activity (PA) is associated with attenuated VC, the precise mechanisms underlying this association, particularly in the MHD population, remain unclear. Irisin, a myokine linked to PA and VC inhibition, is a potential mediator. However, its role in translating PA benefits to reduced VC in MHD patients is unknown.

**Methods:**

This cross-sectional study enrolled 326 patients undergoing maintenance hemodialysis at Xuanwu Hospital Capital Medical University (2020–2023). PA volume was assessed using the International Physical Activity Questionnaire. Serum irisin levels were measured by enzyme-linked immunosorbent assay. Severe VC was defined as an abdominal aortic calcification score (AAC) ≥ 6 on lateral lumbar spine radiography. Multivariable logistic regression and restricted cubic spline models examined the independent and nonlinear associations of PA and irisin with severe VC. Causal mediation analysis estimated the proportion of the PA-VC association mediated by irisin.

**Results:**

Among 326 patients, those in the severe VC group (n=196) exhibited significantly lower PA levels and serum irisin compared to the non-severe group (n=130). In fully adjusted models, both higher PA (odds ratio [OR], 0.51; 95% confidence interval [CI], 0.34-0.74) and higher irisin (OR, 0.56; 95% CI, 0.37-0.83) were independently associated with lower odds of severe calcification, with significant inverse dose-response trends (*P* for trend <0.001). Restricted cubic spline analyses revealed a significant nonlinear relationship between irisin and calcification risk (*P* for nonlinearity = 0.009), while the association for PA suggested a potential plateau at higher activity volumes. These inverse associations were consistent across subgroups stratified by age, gender, Body mass index (BMI), and diabetes status (all interaction *P* > 0.05). Mediation analysis indicated that irisin mediated approximately 60.8% of the total association between PA and severe VC.

**Conclusion:**

This study identifies independent links between physical activity, higher irisin levels, and lower severe vascular calcification risk in hemodialysis patients, with irisin mediating a major part of this association. These findings point to the importance of lifestyle and myokine pathways. Further longitudinal and interventional research is needed to confirm causality and underlying mechanisms.

## Introduction

In patients with chronic kidney disease (CKD), particularly in those with end-stage renal disease undergoing maintenance hemodialysis (MHD), vascular calcification (VC) represents a major clinical burden and a determinant of poor outcomes, with its presence and progression strongly linked to increased cardiovascular and all-cause mortality (1, 2). Its pathogenesis, driven by the advanced uremic milieu, involves the osteogenic transdifferentiation of vascular smooth muscle cells (3–5). Consequently, there is a pressing demand to devise and implement effective prevention and management strategies for this high-risk population.

Given this context, modifiable lifestyle factors, particularly physical activity (PA), have become a major focus of research. Consistent PA is reliably associated with attenuated VC progression in epidemiological and clinical studies, including those involving CKD patients (6–8), potentially through mechanisms such as improved endothelial function and reduced inflammation (9). However, the precise nature of the relationship between PA and VC in MHD patients remains unclear, hindering targeted therapeutic development.

The myokine irisin has emerged as a pivotal molecule potentially linking exercise to vascular health. Beyond its metabolic functions, irisin is recognized as a key mediator in the bone-vascular axis (10, 11). A recent review in this journal comprehensively summarized the experimental evidence that irisin inhibits vascular calcification in cell and animal models (11). However, that review and the existing mechanistic literature have not examined the role of irisin as a mediator of the physical activity–calcification association in humans, nor have they quantified the contribution of this pathway. Experimental evidence suggests it can inhibit VC processes (12). Clinically, lower serum irisin levels are independently associated with increased VC in MHD patients (13, 14), and higher self-reported PA is linked to higher irisin in CKD (15, 16). This suggests a biological connection, yet the interrelationship between PA, irisin, and VC in the unique MHD environment remains to be fully elucidated (17).

Despite these advances, critical knowledge gaps persist. The direct interrelationships between habitual PA, circulating irisin, and VC severity in MHD patients have each been individually linked, yet their combined context remains poorly characterized. More critically, it remains unknown not only whether irisin is associated with the protective effect of PA against VC in this population, but also to what extent any such association might account for the overall relationship between PA and VC. That is, what proportion of the total PA VC association could be statistically accounted for by the irisin pathway? To date, this proportion has never been quantified in any clinical study.

Therefore, we hypothesize that circulating irisin acts as a significant mediator of the protective association between habitual PA and reduced VC in patients undergoing MHD. Testing this hypothesis may provide crucial mechanistic insight, potentially positioning irisin as both a novel biomarker for monitoring lifestyle interventions and a promising therapeutic target.

## Methods

### Study population

**Figure 1:**
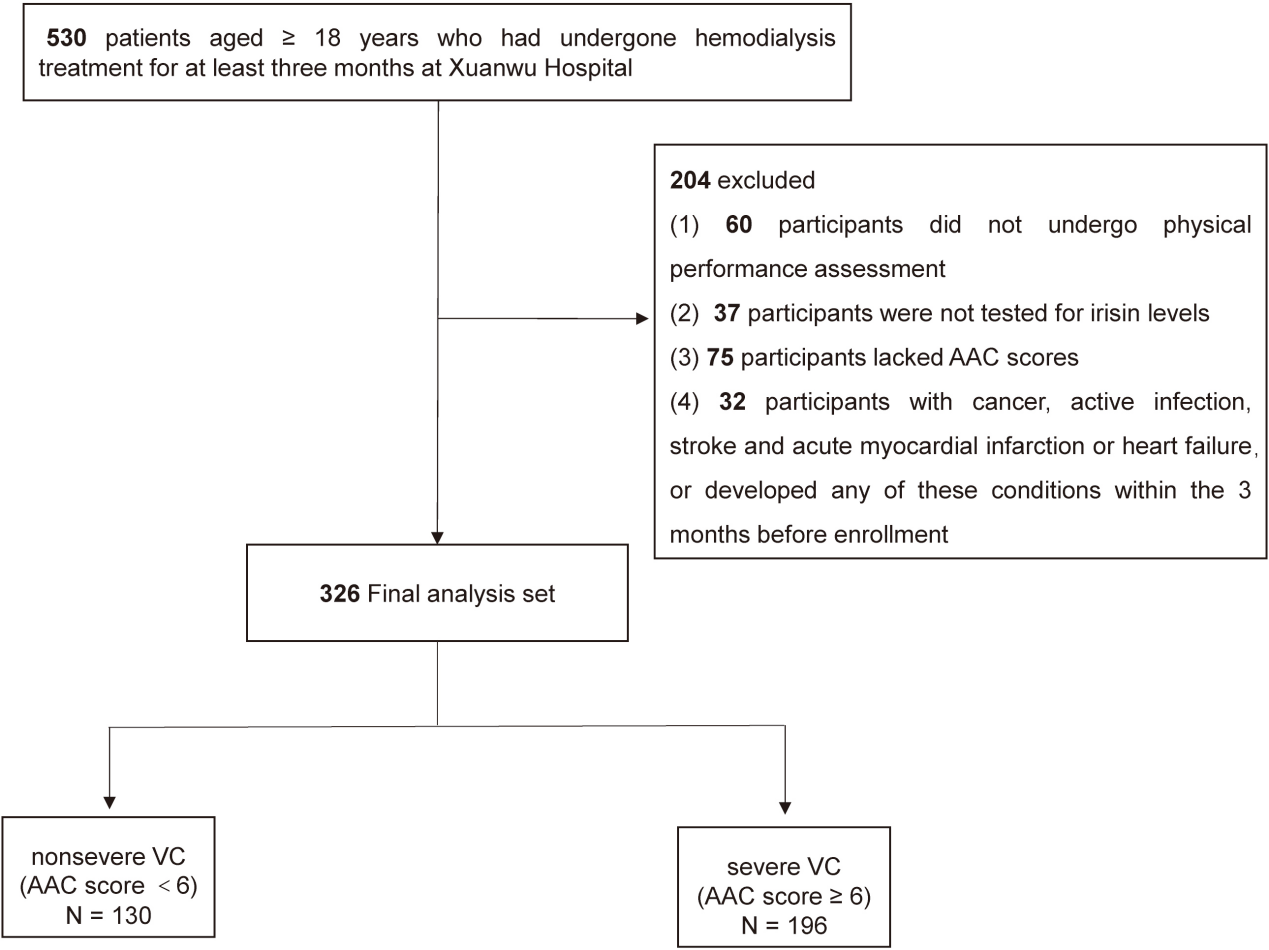
Flow diagram depicting the study flow.

From February 2020 to December 2023, 530 individuals undergoing maintenance hemodialysis at Xuanwu Hospital Capital Medical University were screened, and 326 of them met the criteria for inclusion (shown in **Figure 1**). Patients were eligible when they had been on regular hemodialysis for ≥3 months, were at least 18 years old, and gave written consent to join the research. The exclusion criteria were as follows: (1) inability to complete the International Physical Activity Questionnaire (IPAQ) or missing data from the IPAQ; (2) missing serum irisin levels; (3) missing abdominal aortic calcification (AAC) scores; or (4) patients with a history of cancer, active infection, acute stroke, acute myocardial infarction, or heart failure, or who experienced any of these conditions within 3 months prior to enrollment. The investigation followed the ethical principles set by the Declaration of Helsinki and received approval from the Ethics Committee of Xuanwu Hospital Capital Medical University [approval no. 2019 (131)]. Every participant provided signed consent before participation.

### Data collection and laboratory measurements

Basic demographic and clinical variables were gathered from each participant, including age, sex, stature, body weight, and blood pressure. Pre-dialysis blood pressure was taken while the patient sat upright, using an automated and validated monitor. Resting brachial systolic (SBP) and diastolic blood pressures (DBP) were measured pre-dialysis using a validated, automated electronic sphygmomanometer. Two readings were taken with a brief interval after the participant had rested seated, and the average was recorded for analysis. Routine biochemical parameters, such as albumin, hemoglobin, alkaline phosphatase (ALP), C-reactive protein (CRP), urea, creatinine, uric acid, parathyroid hormone, total protein, calcium, phosphate, fasting blood glucose, potassium, low density lipoprotein (LDL), high density lipoprotein (HDL), triglyceride, total cholesterol, carbon dioxide combining power (CO_2_CP) and ferritin were measured and collected according to standard methods in the laboratory of our hospital. Urea clearance (Kt/V) was calculated using the Daugirdas formula. BMI was calculated as weight/height^2^ (kg/m^2^).

### Measurement of irisin

Fasting serum samples were stored at -80 °C until analysis. Serum irisin levels were measured using an enzyme-linked immunosorbent assay (ELISA) kit (Phoenix Pharmaceuticals, Inc., USA). According to the manufacturer’s instructions. The reported detection limit of the assay is 1.1 ng/mL.

### Physical activity assessment

PA levels over the preceding seven days were quantified with the Chinese adaptation of the long-form International Physical Activity Questionnaire (IPAQ), an instrument with established validity across multiple demographic groups (18). This questionnaire encompasses four principal domains of activity: work, transportation, domestic/gardening, and leisure. For each domain, participants reported the frequency (days/week) and duration (minutes/day) of walking, moderate-intensity, and vigorous-intensity activities. Total PA volume was expressed as metabolic equivalent task minutes per week (MET-min/week), calculated by multiplying the MET values (walking = 3.3 METs, moderate = 4.0 METs, vigorous = 8.0 METs) by the minutes performed per week in each category, and then summing across all domains and intensities.

### Evaluation of abdominal aortic calcification

A radiographic assessment of abdominal aortic calcification (AAC) was performed using lateral lumbar spine images. The extent of calcification was quantified according to the established Kauppila scoring method (19, 20). This system evaluates calcific deposits adjacent to the lumbar vertebrae from L1 to L4. Both the anterior and posterior aortic walls at the midpoint of each intervertebral space are examined independently. A segment score from 0 to 3 is assigned per wall, reflecting the longitudinal length of calcific involvement: 0 indicates no deposits; 1 signifies involvement of less than one-third of the segment; 2 represents one- to two-thirds involvement; and 3 denotes deposits extending beyond two-thirds of the aortic wall. The scores from the anterior and posterior walls across all four vertebral levels are summed, yielding a total AAC score with a potential range from 0 to 24. In this study, using a threshold of AAC score ≥ 6 to indicate severe VC (21), participants were classified into a nonsevere VC group (n=130) and a severe VC group (n=196). All radiographic images were interpreted independently by two assessors blinded to the clinical data of the participants. The interobserver agreement for the total AAC score was excellent, with an intraclass correlation coefficient (ICC) of 0.94.

### Statistical analysis

The Kolmogorov–Smirnov test was applied to examine the distribution of each variable. Values that followed a normal pattern were presented as mean ± standard deviation (SD), while non-normal data and categorical items were reported as frequencies. Independent Student’s t-test and non-parametric tests were used to compare between-group differences. Categorical variables were compared using the Chi-square test.

Multivariable logistic regression analyses were performed to examine the independent associations of PA and serum irisin with severe VC. Three models were constructed: (1) a crude model without covariate adjustment; (2) a model adjusted for baseline age, gender, and BMI; and (3) a fully adjusted model that additionally included the following covariates: for the analysis of PA, serum irisin; for the analysis of irisin, PA; and for both analyses, creatinine, uric acid, dialysis adequacy (Kt/V), alkaline phosphatase (ALP), hemoglobin, albumin, carbon dioxide combining power (CO_2_CP), triglycerides, total cholesterol, diastolic blood pressure (DBP), systolic blood pressure (SBP), calcium, phosphate, and diabetes mellitus. Restricted cubic spline (RCS) modeling was performed to evaluate potential nonlinear dose-response patterns for PA and serum irisin with the odds of having severe VC. Subgroup analyses were conducted by age, gender, BMI, and diabetes status. Interaction terms were included to test for effect modification.

Causal mediation analysis was performed using the lavaan package in R. The model specified physical activity as the exposure, serum irisin as the mediator, and severe vascular calcification (binary) as the outcome, and was estimated using diagonally weighted least squares (WLSMV). The direct, indirect, and total effects were decomposed. Statistical inference for the indirect effect was based on the bootstrap method with 2000 resamples, and bias-corrected and accelerated (BCa) 95% confidence intervals were reported. The number of bootstrap resamples was chosen following established recommendations to ensure stable estimation of the acceleration constant required for BCa intervals. The proportion mediated was calculated as (indirect effect/total effect) × 100%.

All data were analyzed using R software (version 4.3.1; R Foundation for Statistical Computing, Vienna, Austria). A two-sided p < 0.05 indicated statistical significance.

## Results

The analysis finally included 326 maintenance hemodialysis patients, categorized into two groups based on severe VC status (AAC score ≥ 6): a nonsevere VC group (n=130) and a severe VC group (n=196). Their demographic, clinical, and laboratory data are presented in **Table 1**. As anticipated, patients with severe VC were significantly older, had a higher prevalence of diabetes mellitus. The severe VC group exhibited markedly lower levels of PA, measured by the IPAQ score (severe VC: 503.00 [0.00, 1428.00] min/week, nonsevere VC: 1554.00 [952.00, 3682.00] min/week). Concurrently, serum irisin concentrations were significantly reduced in the severe VC group compared to the nonsevere VC group (severe VC: 109.58 [91.79, 115.62] ng/ml, nonsevere VC: 117.65 [113.15, 121.00] ng/ml). Several metabolic and hemodynamic parameters differed notably. Patients with severe VC had higher levels of fasting blood glucose, CRP, and triglyceride, but lower albumin, diastolic blood pressure (DBP) and carbon dioxide combining power (CO_2_CP) (all *P* < 0.05). Other measured parameters related to nutrition, dialysis adequacy, bone-mineral metabolism, and lipid profiles did not differ significantly between the groups.

**Table 1:** Characteristics of study population.

Characteristics	Total N = 326	Nonsevere VC N = 130	Severe VC N=196	P-value
Age (years)	58.07 ± 14.07	51.67 ± 12.83	64.95 ± 11.97	<0.001
Female, n (%)	131 (41)	54 (42)	77 (40)	0.771
BMI (kg/m2)	23.74 ± 3.61	23.66 ± 3.60	23.83 ± 3.63	0.674
DM, n (%)	123 (38)	30 (23)	93 (47)	<0.001
IPAQ scores (min/week)	952.00 [240.00, 2016.00]	1554.00 [952.00, 3682.00]	503.00 [0.00, 1428.00]	<0.001
Serum irisin (ng/ml)	113.15 [102.07, 119.01]	117.65 [113.15, 121.00]	109.58 [91.79, 115.62]	<0.001
AAC scores	5.00 [2.00, 10.00]	1.00 [0.00, 2.00]	9.00 [6.00, 14.00]	<0.001
SBP (mmHg)	135.00 [126.00, 145.00]	138.00 [128.00, 145.00]	134.00 [125.00, 146.00]	0.109
DBP (mmHg)	80.00 [73.00, 87.00]	85.00 [79.00, 91.50]	77.50 [69.00, 85.00]	<0.001
Albumin (g/L)	38.69 ± 4.00	39.35 ± 3.62	37.98 ± 4.28	0.002
Hemoglobin (g/L)	111.97 ± 14.81	112.69 ± 14.51	111.19 ± 15.13	0.363
ALP (U/L)	92.40 ± 57.59	88.00 ± 63.21	97.14 ± 50.63	0.153
CRP (mg/L)	7.54 ± 12.76	5.71 ± 8.68	9.52 ± 15.84	0.007
Urea (mmol/L)	20.19 ± 7.75	20.48 ± 8.10	19.88 ± 7.35	0.488
Creatinine (μmol/L)	873.39 ± 331.85	901.28 ± 374.01	843.37 ± 277.56	0.116
Uric acid (μmol/L)	424.95 ± 89.98	432.33 ± 99.04	417.01 ± 78.61	0.125
Kt/V (single time)	1.62 ± 0.45	1.60 ± 0.45	1.64 ± 0.45	0.519
Parathyroid Hormone (pg/mL)	289.73 ± 273.98	279.72 ± 270.58	300.51 ± 278.04	0.494
Total protein (g/L)	67.69 ± 5.73	67.60 ± 5.89	67.78 ± 5.56	0.77
Calcium (mmol/L)	2.37 ± 0.25	2.35 ± 0.26	2.38 ± 0.24	0.207
Phosphate (mmol/L)	1.72 ± 0.47	1.75 ± 0.50	1.69 ± 0.43	0.256
Fasting blood glucose (mmol/L)	6.91 ± 3.38	6.42 ± 3.12	7.43 ± 3.58	0.007
Potassium (mmol/L)	4.66 ± 0.75	4.73 ± 0.73	4.59 ± 0.77	0.113
LDL (mmol/L)	2.43 ± 0.79	2.48 ± 0.74	2.36 ± 0.83	0.174
HDL (mmol/L)	0.98 ± 0.32	1.00 ± 0.31	0.96 ± 0.32	0.218
Triglyceride (mmol/L)	2.35 ± 1.91	2.11 ± 1.59	2.62 ± 2.18	0.015
Total cholesterol (mmol/L)	4.20 ± 1.13	4.24 ± 1.02	4.15 ± 1.24	0.487
CO_2_CP (mmol/L)	23.92 ± 4.56	24.89 ± 3.63	22.87 ± 5.19	<0.001
Ferritin (ng/mL)	221.60 [93.92, 398.20]	229.85 [86.50, 398.20]	220.65 [98.30, 398.20]	0.916

BMI, body mass index; DM, diabetes mellitus; SBP, Systolic Blood Pressure; DBP, Diastolic Blood Pressure; ALP, alkaline phosphatase; CRP, C-reactive protein; LDL, low density lipoprotein; HDL, high density lipoprotein; CO2CP, carbon dioxide combining power.

Multivariable logistic regression models were employed to assess the independent associations of PA and serum irisin levels with the presence of severe VC. The detailed results are presented in **Table 2** and **Table 3**.

**Table 2:** The Relationship between physical activity and vascular calcification in chronic kidney disease.

Characteristics	Event/n^a^	Model 1		Model 2		Model 3	
		OR (95% CI)	P-value	OR (95% CI)	P-value	OR (95% CI)	P-value
**Physical activity (Continuous^b^)^c^**	–	0.38 (0.27,0.52)	<0.001	0.46 (0.32,0.64)	<0.001	0.51 (0.34,0.74)	<0.001
Physical activity (Tertile)							
T1	109/326	1 [Reference]	NA	1 [Reference]	NA	1 [Reference]	NA
T2	72/326	0.40 (0.21,0.75)	0.004	0.63 (0.31,1.29)	0.21	0.61 (0.27,1.37)	0.23
T3	145/326	0.15 (0.08,0.26)	<0.001	0.22 (0.12,0.40)	<0.001	0.26 (0.13,0.53)	<0.001
**P for trend**	–		<0.001		<0.001		<0.001

Model 1 is the original model, unadjusted for any factor; model 2 is adjusted for baseline age, gender, and BMI; Model 3 is additionally adjusted for Irisin, creatinine, uric acid, Kt/V, ALP, hemoglobin, albumin, CO2CP, triglyceride, total cholesterol, DBP, SBP, Calcium, Phosphate, and diabetes.

^a^Events/N reports the number of events over the total sample size (N = 326) for each tertile.

^b^OR for continuous physical activity corresponds to a 1-unit increase in the activity score.

^c^Row labels in bold indicate the analysis for the continuous exposure variable (physical activity), distinguishing it from the categorical tertile analyses.

OR, odds ratio; CI, confidence interval; NA, not applicable; BMI, body mass index; Kt/V, dialysis adequacy index; ALP, alkaline phosphatase; CO_2_CP, carbon dioxide combining power; DBP, diastolic blood pressure; SBP, systolic blood pressure.

**Table 3:** The Relationship between irisin and vascular calcification in chronic kidney disease.

Characteristics	Event/n^a^	Model 1		Model 2		Model 3	
		OR (95% CI)	P-value	OR (95% CI)	P-value	OR (95% CI)	P-value
**Irisin (Continuous^b^)^c^**	–	0.57 (0.43,0.73)	<0.001	0.56 (0.41,0.74)	<0.001	0.56 (0.37, 0.83)	0.005
Irisin (Tertile)							
T1	108/326	1 [Reference]	NA	1 [Reference]	NA	1 [Reference]	NA
T2	108/326	0.41 (0.23,0.71)	0.002	0.34 (0.17,0.64)	<0.001	0.28 (0.12, 0.63)	0.002
T3	110/326	0.17 (0.09,0.29)	<0.001	0.14 (0.07,0.27)	<0.001	0.11 (0.04, 0.26)	<0.001
**P for trend**	–		<0.001		<0.001		<0.001

Model 1 is the original model, unadjusted for any factor; model 2 is adjusted for baseline age, gender, and BMI; Model 3 is additionally adjusted for PA, creatinine, uric acid, Kt/V, ALP, hemoglobin, albumin, CO2CP, triglyceride, total cholesterol, DBP, SBP, Calcium, Phosphate, and diabetes.

^a^Events/N reports the number of events over the total sample size (N = 326) for each tertile.

^b^OR for continuous irisin corresponds to a 1-unit increase in irisin level (ng/mL).

^c^Row labels in bold indicate the analysis for the continuous exposure variable (irisin), distinguishing it from the categorical tertile analyses.

OR, odds ratio; CI, confidence interval; NA, not applicable; BMI, body mass index; PA, physical activity; Kt/V, dialysis adequacy index; ALP, alkaline phosphatase; CO_2_CP, carbon dioxide combining power; DBP, diastolic blood pressure; SBP, systolic blood pressure.

Regarding PA, a significant independent inverse association was observed. In the fully adjusted model, each incremental unit of PA was associated with substantially lower odds of having severe VC (odds ratio [OR], 0.51; 95% confidence interval [CI], 0.34-0.74; **Table 2**). When analyzed by tertiles of weekly PA duration, which defined as the lowest activity tertile (T1, PA <430.667 min/week), the middle tertile (T2, 430.667 ≤ PA <1428 min/week), and the highest tertile (T3, PA ≥1428 min/week), a clear gradient was apparent. Compared to participants in T1, those in T3 was significantly associated with a reduced risk of having severe VC (OR, 0.26; 95% CI, 0.13-0.53). Whereas participants in T2 did not correlate with a statistically significant risk reduction (*P* = 0.23), trend analysis revealed a significant inverse dose-response relationship between increasing PA levels and the risk of severe VC (*P* for trend < 0.001; **Table 2**).

Similarly, serum irisin levels were assessed in tertiles: T1, irisin<108.75 ng/mL, T2,108.75 ≤ irisin <117.38 ng/mL, and T3, irisin ≥117.38 ng/mL. In the fully adjusted model, compared to the reference T1, both T2 (OR, 0.28; 95% CI, 0.12-0.63; **Table 3**) and T3 (OR, 0.11; 95% CI, 0.04-0.26) were significantly associated with a reduced risk of belonging to the severe VC group. A significant inverse dose-response relationship was observed (*P* for trend <0.001; **Table 3**). Analyzed continuously, each unit increase in irisin was also independently associated with decreased odds of severe VC (OR, 0.56; 95% CI, 0.37-0.83; **Table 3**).

**Figure 2:**
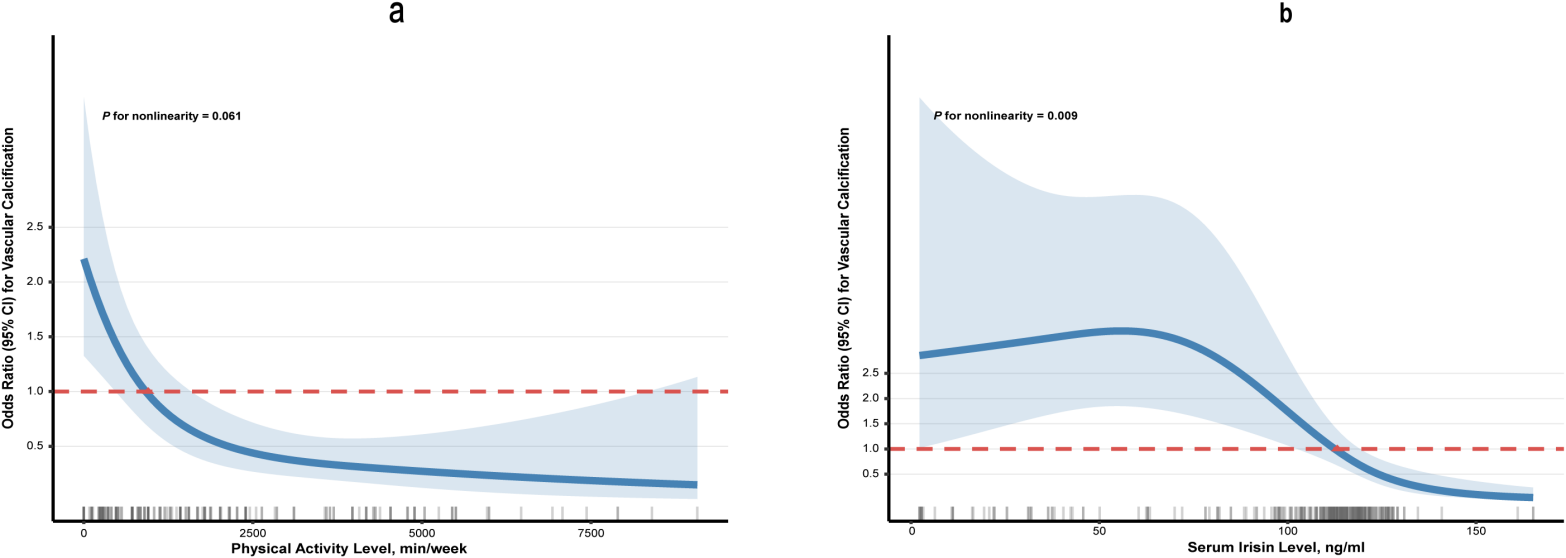
Odds ratios for severe vascular calcification based on restricted cubic spline functions for physical activity levels and serum irisin. **(a)** Nonlinear relationship between physical activity and severe vascular calcification. **(b)** Nonlinear relationship between serum irisin and severe vascular calcification.

Restricted cubic spline (RCS) modeling was performed to evaluate potential nonlinear dose-response patterns for PA and serum irisin with the odds of having severe VC. The results are shown in **Figure 2**.

The association between serum irisin concentration and severe VC risk is displayed in **Figure 1B**. The analysis revealed a markedly nonlinear pattern, which was statistically significant (*P* for nonlinearity = 0.009; **Figure 2b**).

**Figure 2a** presents the relationship between PA level and severe VC risk. The test for nonlinearity did not achieve formal statistical significance (*P* for nonlinearity = 0.061). However, the graphical plot suggests a potential dose-response association. The fitted curve indicates a steep reduction in the odds of severe VC with increasing PA at lower-to-moderate activity volumes. This declining trend appears to attenuate considerably at higher PA levels, implying a plateau in the beneficial association where further increases in activity intensity may yield diminishing marginal returns.

**Figure 3:**
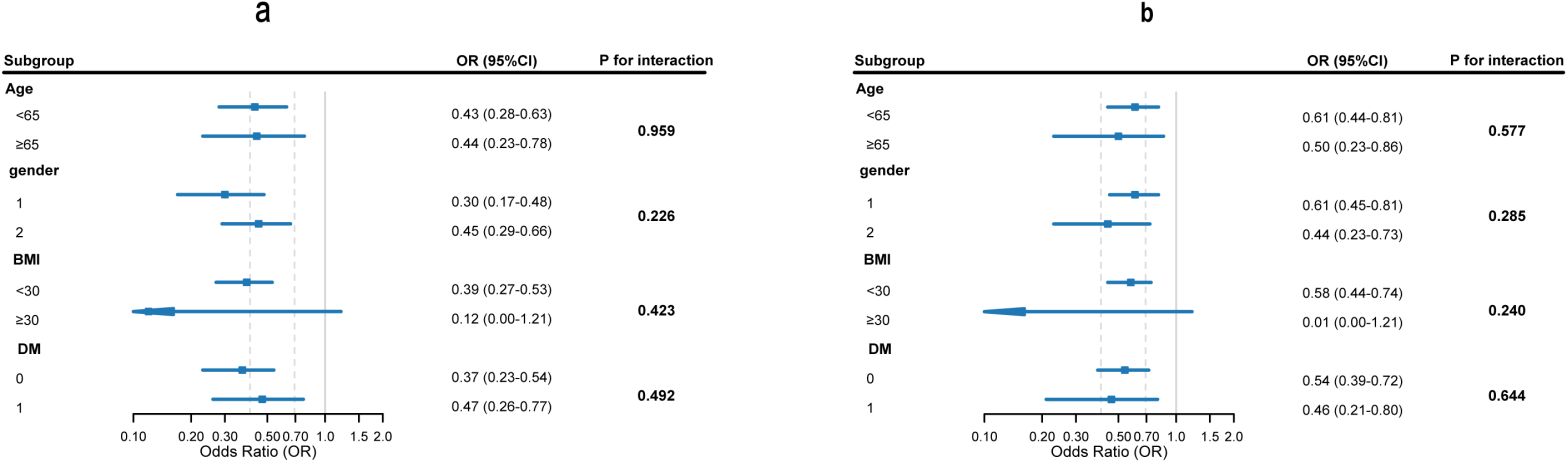
Subgroup analysis of the associations of physical activity **(a)** and serum irisin **(b)** with severe vascular calcification. Forest plots show the odds ratios (with 95% confidence intervals) across subgroups defined by age, gender, BMI, and diabetic status. All associations are adjusted for comprehensive clinical and laboratory covariates.

Subgroup analyses stratified by age, gender, BMI, and diabetes status revealed that higher levels of PA (shown in **Figure 3a**) and higher serum irisin concentrations (shown in **Figure 3b**) were significantly associated with a lower risk of severe VC across all subgroups. No significant heterogeneity in association strength was observed between subgroups (all interaction *P* values > 0.05). Notably, wider confidence intervals were observed in the BMI ≥ 30 kg/m² subgroup, indicating uncertainty in the estimation of association strength within this subgroup.

**Figure 4:**
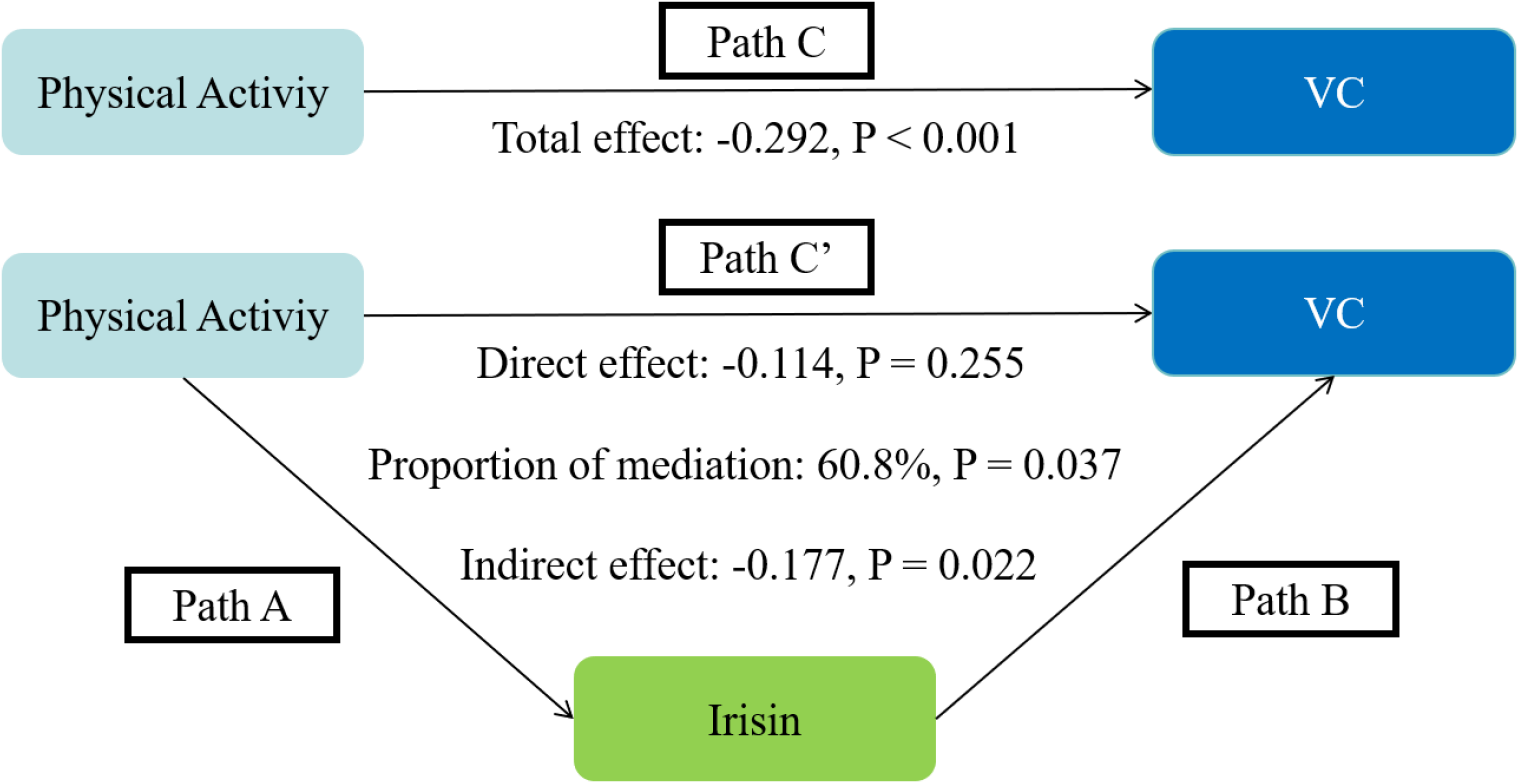
Schematic diagram of the mediation effect analysis. Path C indicates the total effect; path C’ indicates the direct effect. The indirect effect is estimated as the multiplication of paths A and B (path A*B). The mediated proportion is calculated as indirect effect/(indirect effect + direct effect) × 100%. VC, vascular calcification.

To elucidate the potential mediating role of serum irisin, a mediation analysis was conducted. The results indicated a significant positive association between PA and irisin levels (path A: β = 0.231; *P* = 0.017; shown in **Figure 4**). In turn, higher irisin levels were strongly associated with a reduced risk of severe VC (path B: β = -0.766, P < 0.001). The indirect effect through irisin was statistically significant (β = -0.177, P = 0.022), accounting for approximately 60.8% (P = 0.037) of the total effect of PA on VC. The direct effect of PA on VC, after accounting for irisin, was not significant (β = -0.114, P = 0.255).

## Discussion

Based on a cross-sectional investigation of MHD patients, this study explored the interrelationships between PA, serum irisin, and the presence of severe VC. Our analyses revealed two principal findings. First, both higher levels of PA and higher serum irisin concentrations were independently associated with a lower likelihood of severe VC, with dose-response relationships suggesting a nonlinear pattern for each. Second, and more notably, mediation analysis indicated that a substantial proportion of the association between PA and severe VC might operate through serum irisin levels.

This investigation observed independent inverse correlation between serum irisin and severe VC contributes to the evolving understanding of this myokine in vascular health. This finding aligns with some prior observations in MHD populations, supporting the notion that irisin may be involved in the pathophysiology of VC (22). The protective link observed here could be related to irisin’s documented biological functions, such as modulating inflammation and endothelial function (11, 16, 23), furthermore, this association may find a mechanistic explanation in our group’s preclinical work, which suggests the involvement of specific pathways such as AMPK signaling (12).

Beyond established biomarkers, this study provides novel evidence regarding a modifiable lifestyle factor in a specific high-risk population. While inverse associations between PA and VC have been reported in the general population and non-dialysis CKD cohorts, and exercise interventions have been shown to improve bone-mineral metabolism in MHD patients (24–28), data specifically in MHD patients remain scarce and inconclusive. To our knowledge, our cross-sectional study of 326 MHD patients is the first to describe a nonlinear dose-response relationship in this population, and observed an independent inverse association between higher PA levels and a lower likelihood of severe VC. The association was strongest at lower-to-moderate activity levels and appeared to attenuate beyond a certain volume. This pattern, together with the nonlinear trend for irisin, carries clinical implications.

The nonlinear patterns observed carry clinical implications. For serum irisin, the steep initial decline in severe VC risk followed by a plateau suggests a threshold effect: a minimal irisin level may be required to activate anti-calcific pathways such as AMPK-mediated inhibition of osteogenic transdifferentiation, beyond which further increases yield diminishing returns. For physical activity, although the nonlinear trend did not reach statistical significance, the fitted curve indicated a marked risk reduction at low-to-moderate activity volumes that attenuated at higher volumes. This pattern of diminishing returns is clinically reassuring for MHD patients, who often face substantial barriers to exercise. It implies that even modest increases in habitual physical activity—achievable through gentle walking or daily household tasks—could meaningfully reduce vascular calcification risk, without the necessity for high-intensity training. Together, these observations underscore the importance of both PA and irisin as potentially modifiable factors and support a pragmatic, patient-centered approach to lifestyle intervention in this vulnerable population.

Subgroup analyses supported the primary findings, as the inverse associations for both PA and irisin with severe VC were observed across strata of age, gender, BMI, and diabetic status, with no significant interactions detected. This consistency indicates that the observed links might be broadly relevant in MHD patients (29). In the subgroup with obesity (BMI ≥ 30 kg/m²), the association, while imprecise due to a wide confidence interval likely from a smaller sample, showed a direction consistent with the main analysis—a trend requiring confirmation in larger studies.

A key novel insight from this study concerns the mediating function of irisin. Mediation analysis suggested that irisin could account for approximately 60.8% of the total observed association between PA and severe VC. When this irisin-mediated pathway was accounted for, the direct effect of PA was no longer statistically significant. This finding aligns with evidence of a dose-dependent positive association between leisure-time PA and irisin levels observed in the general population (30). These results identify irisin as a principal mediator and suggest a potential mechanism whereby PA could influence vascular health indirectly by elevating irisin secretion, which may then modulate calcification-relevant biological processes. This discovery of a specific myokine-dependent pathway extends the conventional framework for understanding exercise-mediated cardiovascular benefits. Consequently, irisin emerges as a plausible biomarker and a candidate therapeutic target for VC, providing a rationale for future interventions aimed at enhancing irisin activity through tailored exercise or pharmacological agents.

The difference in interpreting the direct effect of PA between regression and mediation models should be acknowledged. This is largely attributable to their distinct methodological approaches. The regression model assesses the overall association, while mediation analysis specifically quantifies the portion explained by a single pathway (31). Rather than conflicting, these results are complementary: the regression confirms the total association of PA with VC status, and the mediation model clarifies that a substantial part of this link operates specifically through irisin.

Several limitations of this investigation must be acknowledged. First, its observational, cross-sectional nature precludes definitive inferences regarding causal relationships; the temporal sequence linking PA, irisin, and the development of severe VC necessitates confirmation via longitudinal studies. Second, PA was assessed using the self-administered IPAQ, a method susceptible to recall inaccuracy and social desirability bias. Third, serum irisin concentration was determined from a single measurement, which cannot reflect its physiological variability over time. Fourth, although extensive covariate adjustment was performed, residual confounding from unmeasured variables, including detailed dietary habits, specific medication regimens, and vitamin D status, may persist (32). Finally, the single-center origin of the cohort limits the generalizability of these findings to broader MHD populations.

In summary, this study first reports independent associations of PA and serum irisin with severe VC in MHD patients and proposes irisin as a significant statistical mediator. These findings highlight the potential importance of both a modifiable lifestyle factor and a related myokine pathway. Future longitudinal and interventional studies are essential to confirm these associations, establish causality, and elucidate the underlying mechanisms.

## Data Availability

The raw data supporting the conclusions of this article will be made available by the authors, without undue reservation.
